# Thalamocortical and intracortical laminar connectivity determines sleep spindle properties

**DOI:** 10.1371/journal.pcbi.1006171

**Published:** 2018-06-27

**Authors:** Giri P. Krishnan, Burke Q. Rosen, Jen-Yung Chen, Lyle Muller, Terrence J. Sejnowski, Sydney S. Cash, Eric Halgren, Maxim Bazhenov

**Affiliations:** 1 Department of Medicine, University of California, San Diego, La Jolla, CA, United States of America; 2 Departments of Radiology and Neurosciences, UCSD, San Diego, CA, United States of America; 3 Computational Neurobiology Lab, Salk Institute for Biological Studies, La Jolla, San Diego, CA, United States of America; 4 Dept. of Neurology, Massachusetts General Hospital and Harvard University, Boston, MA, United States of America; Radboud Universiteit Nijmegen, NETHERLANDS

## Abstract

Sleep spindles are brief oscillatory events during non-rapid eye movement (NREM) sleep. Spindle density and synchronization properties are different in MEG versus EEG recordings in humans and also vary with learning performance, suggesting spindle involvement in memory consolidation. Here, using computational models, we identified network mechanisms that may explain differences in spindle properties across cortical structures. First, we report that differences in spindle occurrence between MEG and EEG data may arise from the contrasting properties of the core and matrix thalamocortical systems. The matrix system, projecting superficially, has wider thalamocortical fanout compared to the core system, which projects to middle layers, and requires the recruitment of a larger population of neurons to initiate a spindle. This property was sufficient to explain lower spindle density and higher spatial synchrony of spindles in the superficial cortical layers, as observed in the EEG signal. In contrast, spindles in the core system occurred more frequently but less synchronously, as observed in the MEG recordings. Furthermore, consistent with human recordings, in the model, spindles occurred independently in the core system but the matrix system spindles commonly co-occurred with core spindles. We also found that the intracortical excitatory connections from layer III/IV to layer V promote spindle propagation from the core to the matrix system, leading to widespread spindle activity. Our study predicts that plasticity of intra- and inter-cortical connectivity can potentially be a mechanism for increased spindle density as has been observed during learning.

## Author summary

The density of sleep spindles has been shown to correlate with memory consolidation. Sleep spindles occur more often in human MEG than EEG recordings. We developed a thalamocortical network model that is capable of spontaneous generation of spindles across cortical layers and that captures the essential statistical features of spindles observed empirically. Our study predicts that differences in thalamocortical connectivity, known from anatomical studies, are sufficient to explain the differences in the spindle properties between EEG and MEG which are observed in human recordings. Furthermore, our model predicts that intracortical connectivity between cortical layers, a property influenced by sleep preceding learning, increases spindle density. Results from our study highlight the role of intracortical and thalamocortical projections on the occurrence and properties of spindles.

## Introduction

Sleep marks a profound change of brain state as manifested by the spontaneous emergence of characteristic oscillatory activities. In humans, sleep spindles consist of waxing-and-waning bursts of field potentials oscillating at 11–15 Hz lasting for 0.5–3 s and recurring every 5–15 s. Experimental and computational studies have identified that both the thalamus and the cortex are involved in the generation and propagation of spindles. Spindles are known to occur in isolated thalamus after decortication *in vivo* and in thalamic slice recordings *in vitro* [1, 2], demonstrating that the thalamus is sufficient for spindle generation. In *in-vivo* conditions, the cortex has been shown to be actively involved in the initiation and termination of spindles [3] as well as the long-range synchronization of spindles [4] [5].

Multiple lines of evidence indicate that spindle oscillations are linked to memory consolidation during sleep. Spindle density is known to increase following training in hippocampal-dependent [6] as well as procedural memory [7] memory tasks. Spindle density also correlates with better memory retention following sleep in verbal tasks [8, 9]. More recently, it was shown that pharmacologically increasing spindle density leads to better post-sleep performance in hippocampal-dependent learning tasks [10]. Furthermore, spindle activity metrics, including amplitude and duration, were predictive of learning performance [11–13], suggesting that spindle event occurrence, amplitude, and duration influence memory consolidation.

In human recordings, spindle occurrence and synchronization vary based on the recording modality. Spindles recorded with magnetoencephalography (MEG) are more frequent and less synchronized, as compared to those recorded with electroencephalography (EEG) [14]. It has been proposed that the contrast between MEG and EEG spindles reflects the differential involvement of the core and matrix thalamocortical systems, respectively [15]. Core projections are focal to layer IV, whereas matrix projections are widespread in upper layers [16]. This hypothesis is supported by human laminar microelectrode data which demonstrated two spindle generators, one associated with middle cortical layers and the other superficial [17]. Taken together, these studies suggest that there could be two systems of spindle generation within the cortex and that these correspond to the core and matrix anatomical networks. However, the network and cellular mechanisms whereby the core and matrix systems interact to generate both independent and co-occurring spindles across cortical layers are not understood.

In this study, we developed a computational model of thalamus and cortex that replicates known features of spindle occurrence in MEG and EEG recordings. While our previous efforts have been focused on the neural mechanisms involved in the generation of isolated spindles[5], in this study we identified the critical mechanisms underlying the spontaneous generation of spindles across different cortical layers and their interactions.

## Results

### Spindle occurrence is different in EEG and MEG

**Fig 1:**
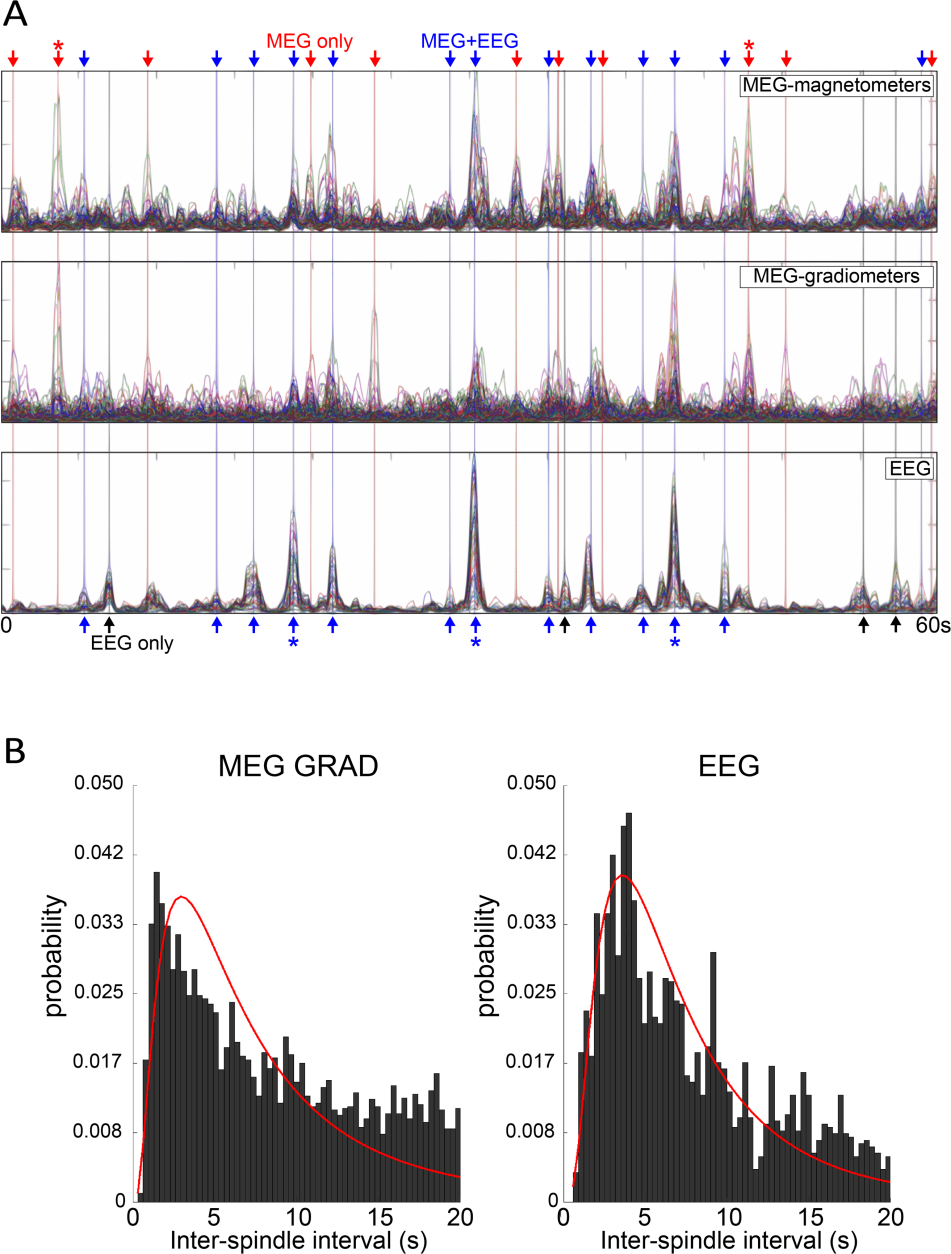
Experimental observations. (A) Envelopes of spindle-band (6-15Hz) activity in human MEG magnetometers, MEG gradiometers, and EEG. Within-modality channels are superimposed. Arrows indicate spindles that occur in MEG only, EEG only, and both MEG & EEG, in red, black, and blue, respectively. Note that EEG spindles are less frequent and have greater synchrony than MEG spindles. (B) Histograms of the inter-spindle intervals during stage 2 sleep show a non-normal distribution in the occurrence of spindles in both the MEG gradiometer and EEG channels. Compare with simulated data in Fig 4B.

Histograms of EEG and MEG gradiometer inter-spindle intervals are shown in Fig 1C. For neither channel type are ISIs distributed normally as determined by Lilliefors tests (D_2571_ = 0.1062, p = 1.0e-3, D_4802_ = 0.1022, p = 1.0e-3), suggesting that traditional descriptive statistics are of limited utility. However, the ISI at peak of the respective distributions is longer for EEG than it is the MEG. In addition, a two-sample Kolmogorov-Smirnov test confirms that EEG and MEG ISIs are not drawn from the same distribution (D_2571,4802_ = 0.079, p = 1.5e-9). While the data where not found to be drawn from any parametric distribution with 95% confidence, an exponential fit (MEG) and lognormal fit (EEG) are shown in red overlay for illustrative purposes. These data are consistent with previous empirical recordings [18] and suggest that sleep spindles have different properties across superficial vs. deep cortical layers.

### Frequent local spindles in the core and rare global spindles in the matrix

**Fig 2:**
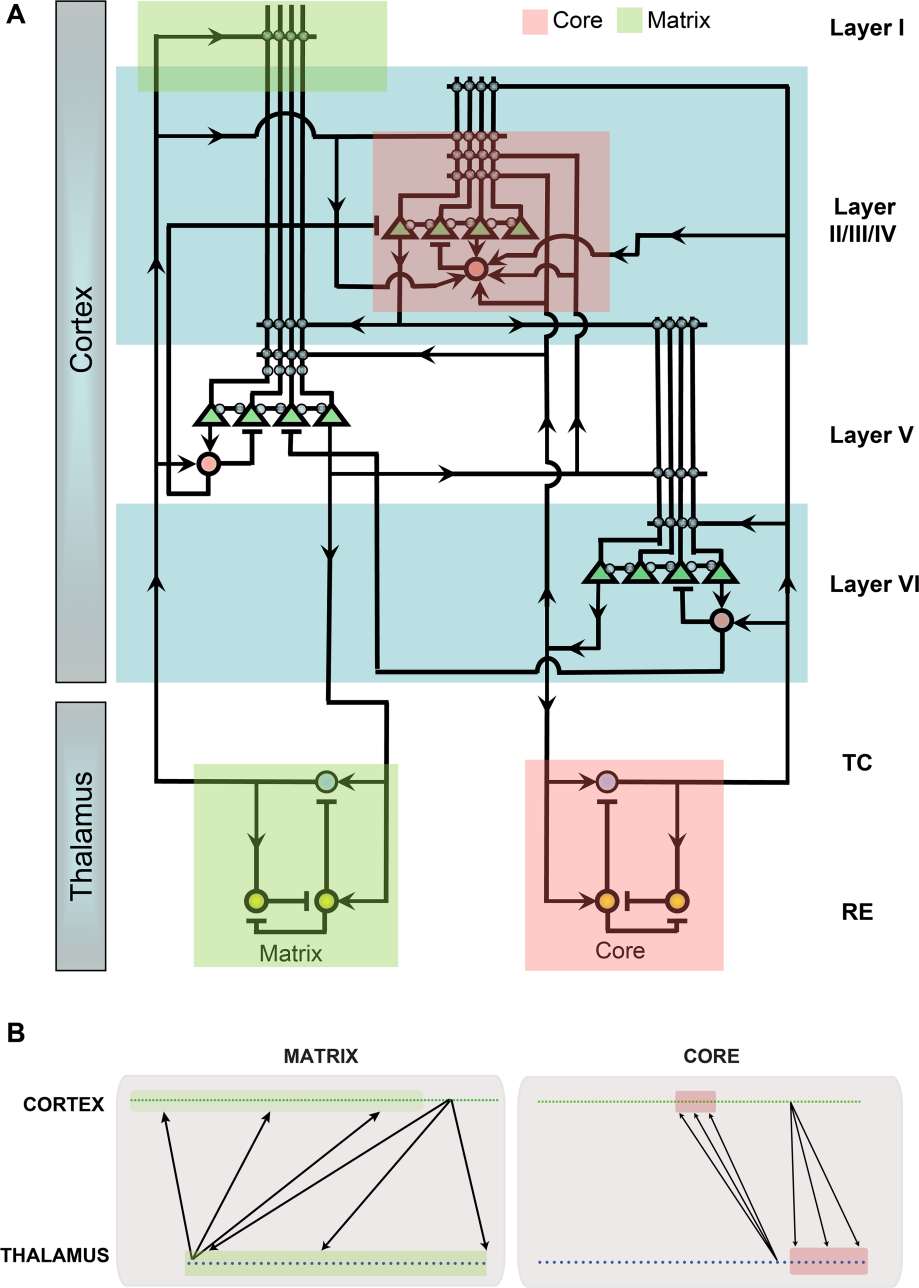
Connectivity in the thalamocortical network model. A. The network architecture included three one-dimensional network layers each containing cortical pyramidal neurons and inhibitory interneurons and two thalamic neuronal groups corresponding to the matrix and core systems. Green triangles indicate cortical pyramidal neurons, red circles indicate inhibitory interneurons, blue circles indicate thalamic relay neurons and yellow circles indicate thalamic reticular neurons. Lines ending with arrows indicate excitatory AMPA/NMDA connections while lines ending with bars indicate inhibitory GABAergic connections. B. Schematic connectivity for differences in the spatial extent of thalamocortical and corticothalamic projections for the core and matrix systems.

To investigate the mechanisms behind distinct spindle properties across cortical locations as observed in EEG and MEG signals, we constructed a model of thalamus and cortex that incorporated the two characteristic thalamocortical systems: core and matrix. These systems contained distinct thalamic populations that projected to the superficial (matrix) and middle (core) cortical layers. Four cell types were used to model distinct cell populations: thalamocortical relay (TC) and reticular (RE) neurons in the thalamus, and excitatory pyramidal (PY) and inhibitory (IN) neurons in each of three layers of the cortical network. A schematic representation of the synaptic connections and cortical geometry of the network model is shown in Fig 2. In the matrix system, both thalamocortical (from matrix TCs to the apical dendrites of layer 5 pyramidal neurons (PYs) located in the layer 1) and corticothalamic synapses (from layer 5 PYs back to the thalamus) formed diffuse connections. The core system had a focal connection pattern in both thalamocortical (from core TCs to PYs in the layer III/IV) and corticothalamic (from layer VI PYs to the thalamus) projections. Because spindles recorded in EEG signal reflect the activity of superficial layers while MEG records spindles originating from deeper layers (Fig 1 and [19]), we compared the activity of the model’s matrix system, which has projections to the superficial layers, to empirical EEG recordings and compared the activity in model layer 3/4 to empirical MEG recordings.

**Fig 3:**
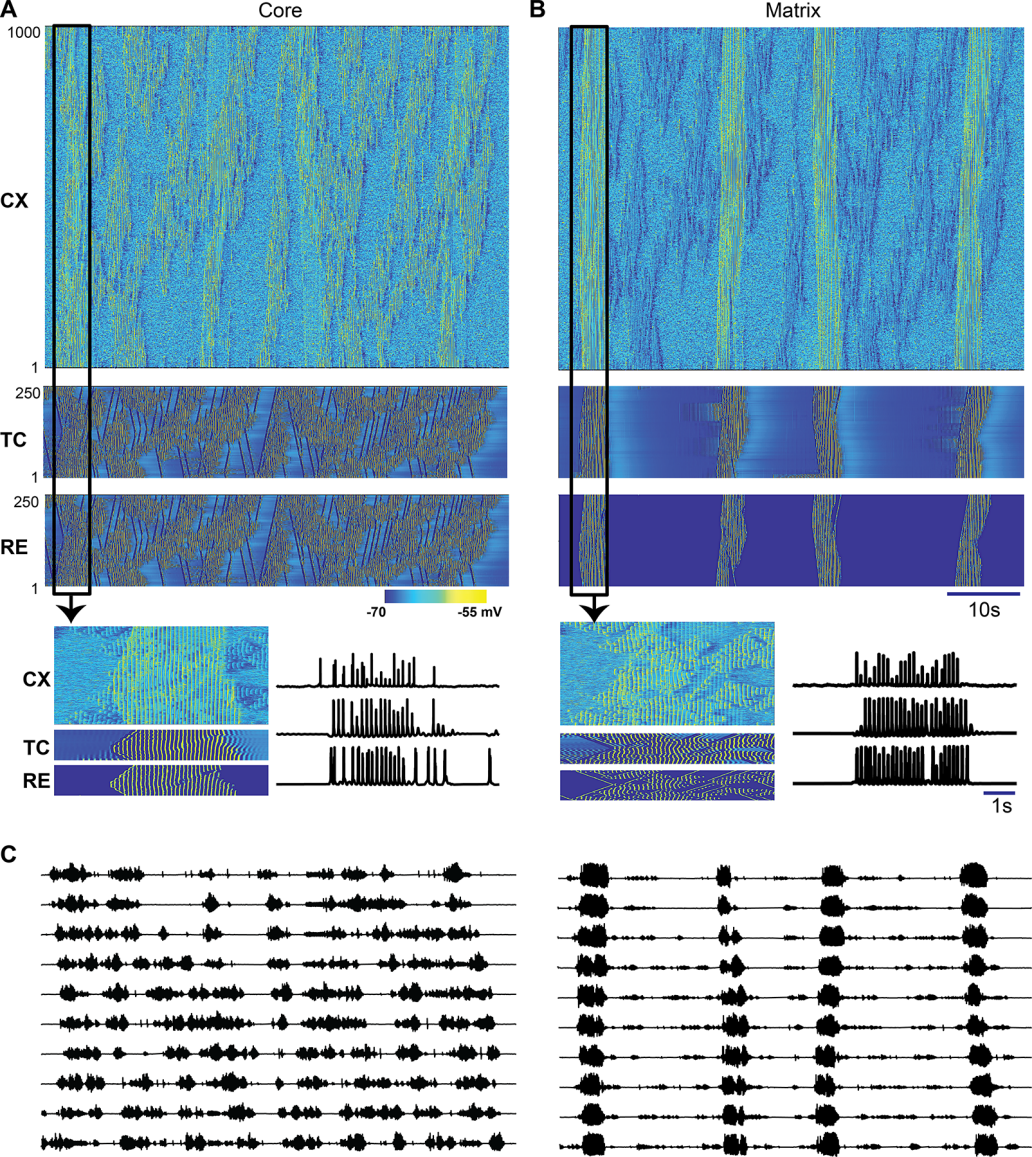
Spindle activity is different in the core and matrix systems. (A, B) Space-time plots of activity in the thalamocortical network including the core system (A) and matrix system (B). (C) Average network activity (simulated LFP) filtered between 6–15 Hz was measured from 10 non-overlapping locations (each group contains 100 PYs) of the cortical network. Note, spindles occur more frequently (but less synchronously) in L3/4 (left) and less frequent (but more synchronous) in L5 (right).

In agreement with our previous studies [3, 5, 20, 21], simulated stage 2 sleep consisted of multiple spindle events involving thalamic and cortical neuronal populations (Fig 3). During one such typical spindle event (highlighted by the box in Fig 3A and 3B), cortical and thalamic neurons in both the core and matrix system had elevated and synchronized firing (Fig 3A bottom), consistent with previous in-vivo experimental recordings [22]. In the model, spindles within each system were initiated from spontaneous activity within cortical layers and then spread to thalamic neurons, similar to our previous study[5]. The spontaneous activity due to miniature EPSPs in glutamergic cortical synapses led to fluctuations in membrane voltage and sparse firing. At random times, the miniature EPSPs summed such that a small number of locally connected PY neurons spiked within a short window (<100ms), which then induced spiking in thalamic cells through corticothalamic connections. This initiated spindle oscillations in the thalamic population mediated by TC-RE interactions as described before [20, 23, 24]. Thalamic spindles in turn propagated to the neocortex leading to joint thalamocortical spindle events whose features were shaped by the properties of thalamocortical and corticothalamic connections. In this study, we examined how the process of spindle generation occurs in a thalamocortical network with mutually interacting core and matrix systems, wherein the thalamic network of each system is capable of generating spindles independently.

Based on the anatomical data [16], the main difference between the modeled core and matrix systems was the radii or fanout of connections in thalamocortical and corticothalamic projections (in the baseline model, the fanout was 10 times wider for the matrix compared to the core system). Furthermore, the strength of each synaptic connection was scaled by the number of input connections to each neuron [25, 26], leading to weaker individual thalamocortical projections in the matrix as compared to the core. These differences in the strength and fanout of thalamocortical connectivity resulted in distinctive core and matrix spindle properties (see Fig 3A, right vs left). First, both cortical and thalamic spindles were more spatially focal in the core system as only a small subset of neurons was involved in a typical spindle event at any given time. In contrast, within the matrix system spindles were global (involving the entire cell population) and highly synchronous across all cell types. These results are consistent with our previous studies [5] and suggest that the connectivity properties of thalamocortical projections determine the degree of synchronization in the cortical network. Second, spindle density was higher in the core system compared to the matrix system. At every spatial location in the cortical network of the core system, the characteristic time between spindles was shorter compared to that between spindles in the matrix system (Fig 3A left vs right). In order to quantify the spatial and temporal properties of spindles, we computed an estimated LFP as an average of the dendritic synaptic currents for every group of contiguous 100 cortical neurons. LFPs of the core system were estimated from the currents generated in the dendrites of layer 3/4 neurons while the LFP of the matrix system was computed from the dendritic currents of layer 5 neurons, located in the superficial cortical layers (Fig 2). After applying a bandpass filter (6–15 Hz), the spatial properties of estimated core and matrix LFP (Fig 3C) closely matched the MEG and EEG recordings, respectively (Fig 1). In subsequent analyses, we used this estimated LFP to further examine the properties of the spindle oscillations in the core and matrix systems.

### Spindle occurrence can be captured by a non-periodic process

**Fig 4:**
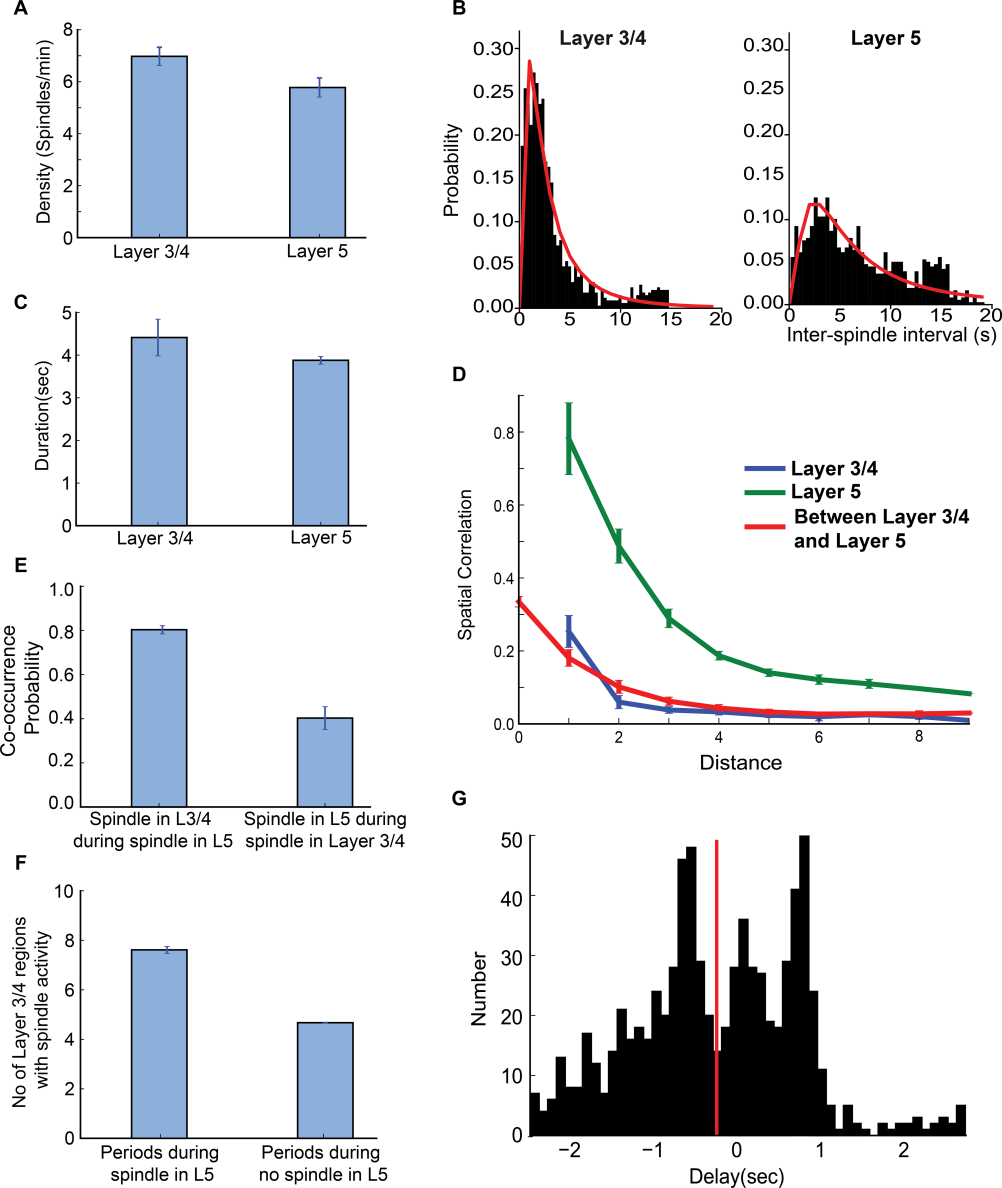
Statistical proprieties of sleep spindles in the core and matrix systems. (A) Spindle density measured from the filtered averaged network activity in the core and matrix systems, with error bars indicating standard error across different network locations. (B) Histograms of inter-spindle intervals reveal a non-normal distribution in the occurrence of spindles in both the core and matrix systems. (C) Spindle duration as measured by the onset to offset of spindle that was identified by a threshold measure (see method section for details). (D) Spatial correlation within and between layers measured as a function of the distance between network sites. (E) Probability of co-occurrence of spindle in core and matrix system. (F) Probability of spindle co-occurrences in the core and matrix systems. (G) Delay between the onset of spindles in the core and matrix, negative values indicate that the spindle in the core precedes that in the matrix.

We identified spindles in the estimated LFP using an automated spindle detection algorithm similar to that used in experimental studies (details are provided in the method section). The spindle density, defined as the number of spindles occurring per minute of simulation time, was greater in the core compared to the matrix (Fig 4A) as confirmed by an independent-sample t-test (t(18) = 7.06, p<0.001 for across estimated LFP channels and t(2060) = 19.2, p<0.001 across all spindles). The results of this analysis agree with the experimental observation that MEG spindles occur more frequently than EEG spindles. While the average spindle density was significantly different between the core and matrix, in both systems the distribution of inter-spindle intervals peaks below 4 seconds and has a long tail (Fig 4B). A two sample KS test comparing the distributions of inter-spindle intervals confirmed that the intervals were derived from different distributions (D_1128,932_ = 0.427, p<0.001). The peak ISI of the core was shorter than that of the matrix system, suggesting that the core network experiences shorter and more frequent quiescence periods than the matrix population. Furthermore, maximum-likelihood fits of the probability distributions (red line in Fig 4B) confirmed that the intervals of spindle occurrence cannot be described by a normal distribution. The long tails of the distributions suggest that a Poisson like process, as oppose to a periodic process, is responsible for spindle generation. This observation is consistent with previous experimental results [18, 27] and suggests that our computational model replicates essential statistical properties of spindles observed in *in vivo* experiments.

Several other features of simulated core and matrix spindles were similar to those found in experimental recordings. The average spindle duration was significantly higher in the core compared to the matrix system (Fig 4C) as confirmed by independent-sample t-test (t(2060) = 16.3, p<0.001). To quantify the difference in the spatial synchrony of spindles between the core and matrix systems, we computed the spatial correlation [28] between LFP groups at different distances (measured by the location of a neuron group in the network). The correlation strength decreased with distance for both systems (Fig 4D). However, the spindles in the core system were less spatially correlated overall when compared to spindles in the matrix system.

### Co-occurring core and matrix spindles recruit large cortical regions in the core system through recurrent excitation between layers

Simultaneous EEG and MEG measurements have found that about 50% of MEG spindles co-occur with EEG spindles, while about 85% of EEG spindles co-occur with MEG spindles [29]. Further, a spindle detected in the EEG signal is found to co-occur with about 66% more MEG channels than a spindle detected in MEG. Our model generates spindling patterns consistent with these features. The co-occurrence probability revealed that during periods of spindles in the matrix system, there was about 80% probability that core was also generating spindles (Fig 4E). In contrast, there was only a 40% probability of observing a matrix spindle during a core system spindle. An independent-sample t-test confirmed this difference between the systems across estimated LFP channels (t(14) = 31.4, p<0.001). Furthermore, we observed that the number of LFP channels that were simultaneously activated during a spindle event in the core system was higher when a spindle co-occurred in the matrix versus times when the spindles only occurred in the core (Fig 4F, t(14) = 67.2, p<0.001). This suggests that the co-occurrences of spindles in both systems are rare events but lead to the wide spread activation in both the core and matrix when they take place.

Finally, we examined the delay between spindles in the core and matrix systems (Fig 4G). We observed that on average (red line in Fig 4G), the spindle originated from the core system then spread to the matrix system with a mean delay of about 300 ms (delay was measured as the difference in onset times between co-occurring spindles within a window of 2,500 ms; negative delay values indicate spindles in which the core preceded matrix). The peak at -750 ms corresponds to spindles originating from the core system, while the peak at +750 ms suggests that at some network sites, spindles originated in the matrix system and then spread to the core system. While there were almost no events in which the matrix preceded the core by more than 1 sec (right of Fig 4G), many events occurred in which the core preceded the matrix by more than 1 sec (left of Fig 4G).

In sum, these results suggest that spindles were frequently initiated locally in the core system, then propagate to and spread throughout the matrix system. This can trigger spindles at the other locations of the core, so eventually, even regions in the core system that were not previously involved become recruited. These findings explain the experimental result that spindles are observed in more MEG channels when they also co-occur in the EEG [29].

### Impact of thalamocortical and corticothalamic connections on spindle occurrence

We leveraged our model to examine factors that may influence spindle occurrence across cortical layers. The main difference between the core and matrix systems in the model was the breadth or fanout of the thalamic projections to the cortical network. Neuroanatomical studies suggest that the core system has focused projections while matrix system projects widely [16]. Here, we assessed the impacts of this characteristic by systematically varying the connection footprint of the thalamic matrix to superficial cortical regions, while holding the fanout of the thalamic core to layer 3/4 projections constant. We also modulated the corticothalamic projections in proportion to the thalamocortical projections. Using the estimated LFP from the cortical layers corresponding to core and matrix system, respectively, we quantified various spindle properties as the fanout was modulated.

**Fig 5:**
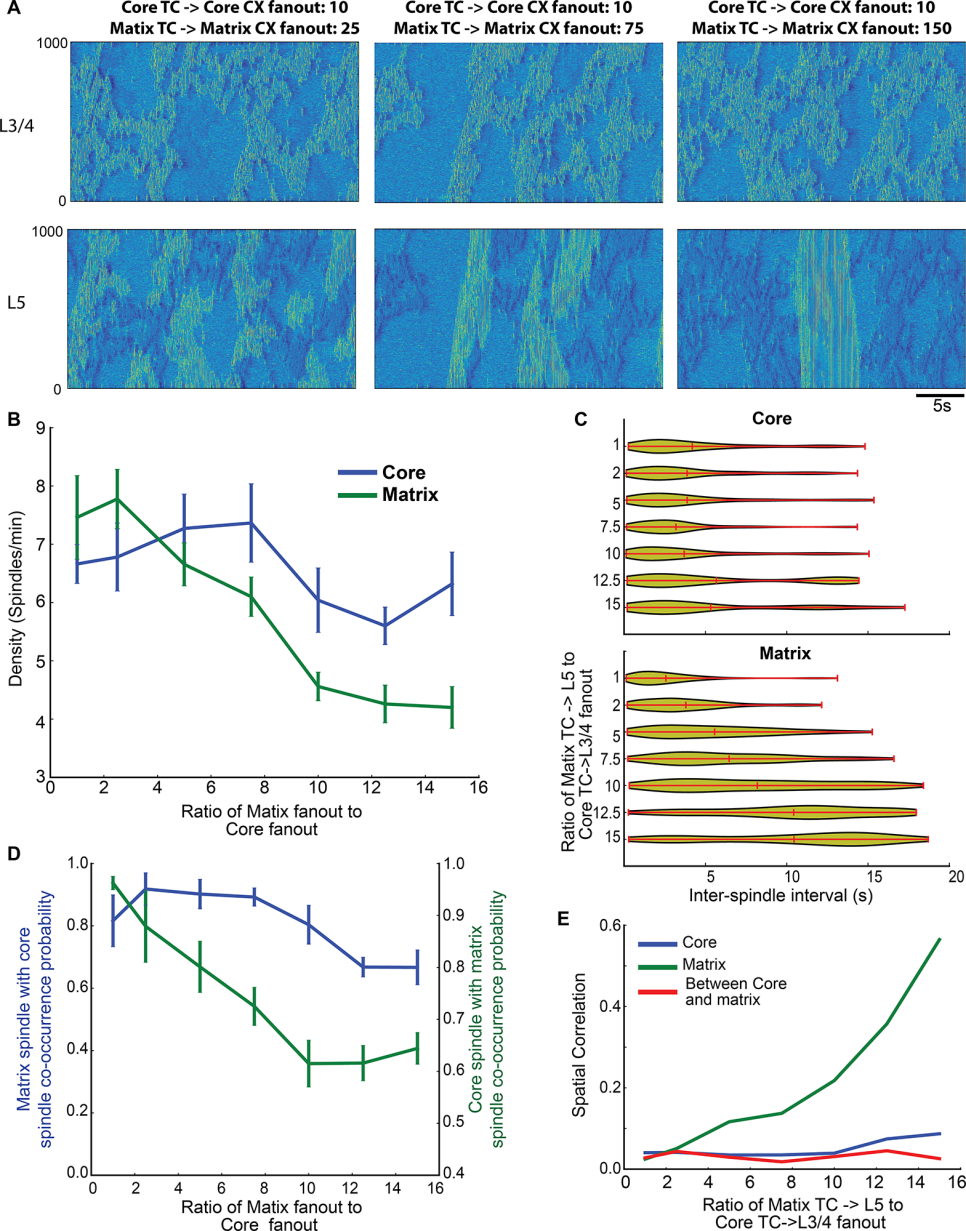
Effect of thalamocortical connectivity on spindle properties. (A) Space-time plots of network activity in cortical layers for different fanout of the thalamocortical and corticothalamic connections. (B) Spindle density for different fanout conditions. The X-axis shows the ratio of thalamic projection fanout in the matrix system to that in the core system. The actual thalamocoritcal and corticothalamic radii for the different ratios (1,2.5, 5, 7.5, 10, 12.5, 15) were 10/2/10/2 (core TC->L3/4, L6->core TC, matrix TC->L5, L5->matrix TC), 10/2/25/5, 10/2/50/10, 10/2/75/15, 10/2/100/20, 10/2/125/25, 10/2/150/30. (C) Violin plots showing the distribution of inter-spindle intervals as a function of thalamocortical fanout. (D, E) Probability of spindle co-occurrence between core and matrix, and spatial correlation as a function of synaptic fanout.

Spindle density (the number of spindles per minute) in both layers was sensitive to the matrix system’s fanout. ANOVA confirmed significant effects of fanout and layer location, as well as an interaction between layer and fanout (fanout: F(6,112) = 66.4; p<0.01, Layer: F(1,112) = 65.18; p<0.01 and interaction F(6,112) = 22.8; p<0.01). When the matrix and core thalamus had similar fanouts (ratio 1 and 2.5 in Fig 5B), we observed a slightly higher density of spindles in the matrix than in the core system. This observation is consistent with the properties of these circuits (see Fig 2), wherein the matrix system contains direct reciprocal projections connecting cortical and thalamic subpopulations and the core system routes indirect projections from cortical (layer III/IV) neurons through layer VI to the thalamic nucleus. When the thalamocortical fanout of the matrix system was increased to above ~5 times the size of the core system, the density of spindles in the matrix system was reduced to around 4 spindles per minute. Interestingly, the density of spindles in the core system was also reduced when the thalamocortical fanout of the matrix system was further increased to above ~10 times of that in the core system (ratio above 10 in Fig 5B). This suggests that spindle density in both systems is determined not only by the radius of thalamocortical vs. corticothalamic projections, but also by interactions between the systems among the cortical layers. We further expound on the role of these cortical connections in the next section.

We also examined the effect of thalamocortical fanout on the distribution of inter-spindle intervals (Fig 5C). Although the mean value was largely independent of the projection radius, a long tailed distribution was observed for all values of fanout in the core. Contrastingly, in the matrix system the mean and peak of the inter-spindle interval shifted to the right (longer intervals) with increased fanout. With large fanouts, the majority of matrix system spindles had very long periods of silence (10-15s) between them. This suggests that thalamocortical fanout determines the peak of the inter-spindle interval distribution, but does not alter the stochastic nature of spindle occurrence.

The degree of thalamocortical fanout also influenced the co-occurrence of spindles in the core and matrix systems (Fig 5D). Increasing the fanout of the matrix system reduced spindle co-occurrence between two systems. This reduction resulted mainly from lower spindle density in both layers. However, the co-occurrence of core spindles during matrix spindles was higher for all values of fanout when matrix thalamocortical projections were at least 5 times broader than core projections. This suggests that the difference in spindle co-occurrence between EEG and MEG as observed in experiments [14] depends mainly on the difference in the radius of thalamocortical projections between the core and matrix systems, while overall level of co-occurrence is determined by the interaction between cortical layers.

We examined how spatial correlations during periods of spindles vary depending on the fanout of thalamocortical projections. The spatial correlation quantifies the degree of synchronization in the estimated LFP signals of network locations as a function of the distance between them. As expected, increasing the distance reduced the spatial correlation (Fig 4D). We next measured the mean value of the spatial correlation for each fanout condition. The mean correlation increased as a function of the fanout in the matrix system (Fig 5A). However, the spatial correlation within the core, and between the core and matrix systems, did not change with increases in the fanout, suggesting that the spatial synchronization of core spindles is largely influenced by thalamocortical fanout but not by interactions between the core and matrix systems as was observed for spindle density.

### Feedforward input to the matrix critically determines spindle density

**Fig 6:**
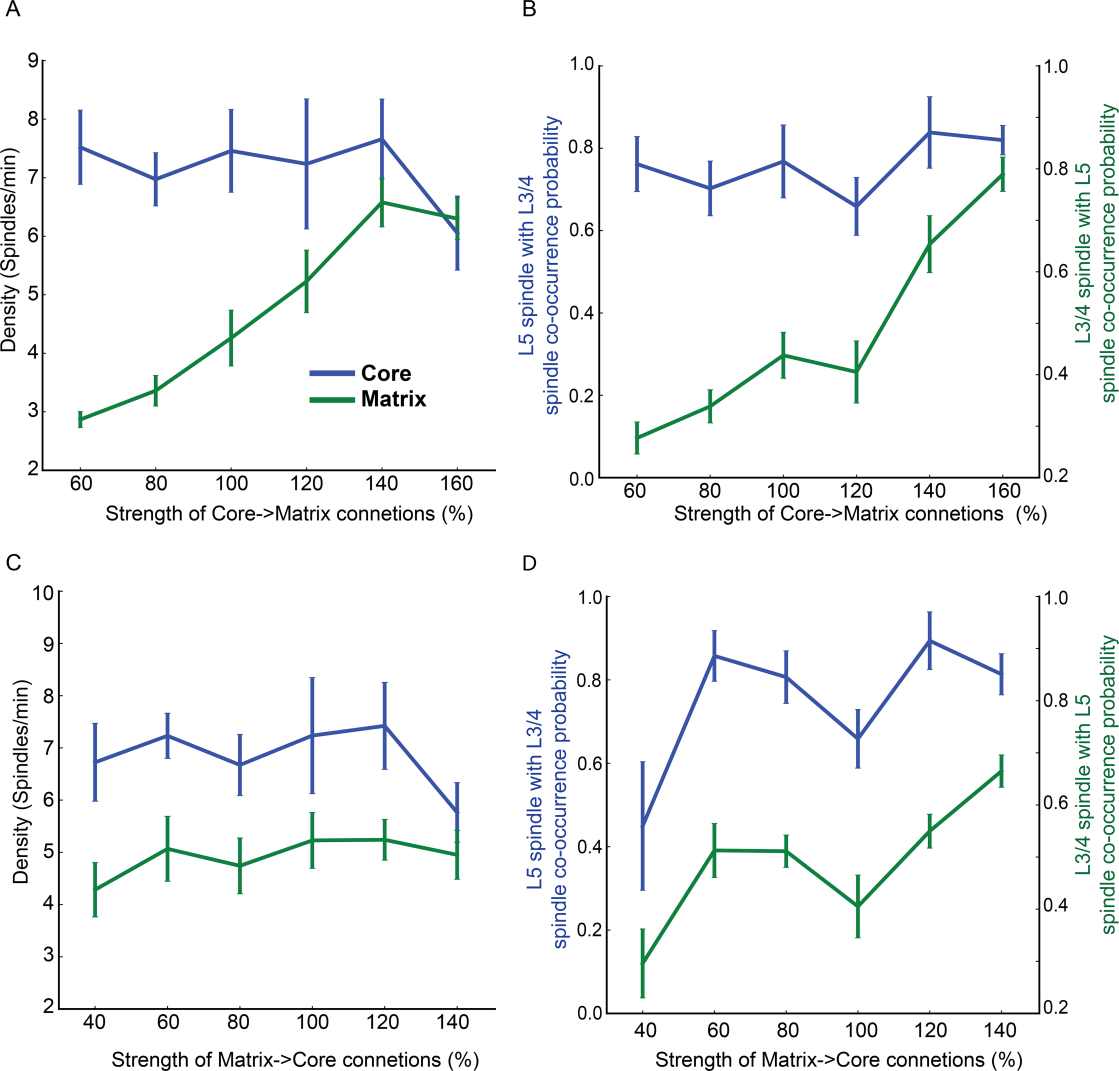
Effect of interlaminar connectivity on spindle properties. (A,B) Spindle density and probability of co-occurrence between the core and matrix as a function of strength of the AMPA and NMDA connections from L3/4 to L5. The value of 100% interlaminar connectivity corresponds to half of the intralaminar connectivity strength (within L3/4 and L5). (C, D) Spindle density and probability of co-occurrence as a function of the AMPA and NMDA connection strength from L5 to L3/4 neurons.

Does intra-cortical excitatory connectivity between layer 3/4 of the core system and layer 5 of the matrix system affect spindle occurrence? To answer this question, we first varied the strength of excitatory connections (AMPA and NMDA) from the core to matrix pyramidal neurons (Fig 6A and 6B). Here the reference point (or 100%) corresponds to the strength used in previous simulations, i.e. half the strength of a within-layer connection. The spindle density varied with the strength of the interlaminar connections (Fig 6A). For low connectivity strengths (below 100%), the spindle density of the matrix system was reduced significantly, while at high strengths (above 140%) the matrix system spindle density exceeded that of the control (100%). There were significant effects of connection strength and layer on the spindle density, as well as an interaction between the two factors (connection strength: F(5,96) = 24.7; p<0.01, layer: F(5,96) = 386.6; p<0.01 and interaction F(5,96) = 36.9; p<0.01) that suggests a layer-specific effect of modulating excitatory interlaminar connection strength. Similar to the spindle density, spindle co-occurrence between the core and matrix systems also increased as a function of interlaminar connection strength, reaching 80% for the both core and matrix at 150% connectivity. In contrast, changing the strength of excitatory connections from layer 5 to layer 3/4 had little effect on the spindle density, (Fig 6C). Taken together, these results suggest that the strength of the cortical core-to-matrix excitatory connections is one of the critical factors in determining spindle density and co-occurrence among spindles across both cortical lamina and the core/matrix systems.

## Discussion

Using computational modeling and data from EEG/MEG recordings in humans we found that the properties of sleep spindles vary across cortical layers and are influenced by thalamocortical, corticothalamic and cortico-laminar connections. This study was motivated by empirical findings demonstrating that spindles measured in EEG have different synchronization properties from those measured in MEG [14, 29]. EEG spindles occur less frequently and more synchronously in comparison to MEG spindles. Our new study confirms the speculation that anatomical differences between the matrix thalamocortical system, which has broader projections that target the cortex superficially, and the core system, which consists of focal projections which target the middle layers, can account for the differences between EEG and MEG signals. Furthermore, we discovered that the strength of corticocortical feedforward excitatory connections from the core to matrix neurons determines the spindle density in the matrix system, which predicts a specific neural mechanism for the interactions observed between MEG and EEG spindles.

There were several novel findings in this study. First, we developed a novel computational model of sleep spindling in which spindles manifested as a rare but global synchronous occurrence in the matrix pathway and a frequent but local occurrence in the core pathway. In other words, many spontaneous spindles occurred locally in the core system but only occasionally did this lead to globally organized spindles appearing in the matrix system. As a result, only a fraction of spindles co-occurred between the pathways (about 80% in matrix and 40% in core pathway). This is consistent with data reported for EEG vs MEG *in vivo* (Fig 1). In contrast, in our previous models [3, 5], spindles were induced by external stimulation and always occurred simultaneously in the core and matrix systems, but with different degrees of internal synchrony. In addition, these studies did not examine how the core and matrix pathways interact during spontaneously occurring spindles. Second, in this study we found that the distribution of the inter-spindle intervals between spontaneously occurring spindles in both the core and matrix pathways had long tails similar to a log-normal distribution. This result is consistent with analyses of MEG and EEG data reported in this study and in our prior study [18]. In our previous models [3, 5], spindles were induced by external stimulation and the statistics of spontaneously occurring spindles could not be explored. Third, we demonstrated that the strength of thalamocortical and corticothalamic connections determined the density and occurrence of spontaneously generated spindles. The spindle density was higher in the core pathway as compared to the matrix pathway with high co-occurrence of core spindles with matrix spindles. These findings were corroborated with experimental evidence from EEG/MEG recordings. Finally, we reported that laminar connections between the core and matrix could be a significant factor in determining spindle density, suggesting a possible mechanism of learning. When the strength of these connections was increased in the model, there was a significant increase in spindle occurrence, similar to the experimentally observed increase in spindle density following recent learning [10].

The origin of sleep spindle activity has been linked to thalamic oscillators based on a broad range of experimental studies [2, 30, 31]. The excitatory and inhibitory connections between thalamic relay and reticular neurons are critical in generating spindles [20, 23, 32, 33]. However, in intact brain, the properties of sleep spindles are also shaped by cortical networks. Indeed, the onset of a spindle oscillation and its termination are both dependent on cortical input to the thalamus [3, 34, 35]. In model studies, spindle oscillations in the thalamus are initiated when sufficiently strong activity in the cortex activates the thalamic network, and spindle termination is partially mediated by the desynchronization of corticothalamic input towards the end of spindles [3,32]. However, in simultaneous cortical and thalamic studies in humans, thalamic spindles were found to be tightly coupled to a preceding downstate, which in turn was triggered by converging cortical downstates [36]. Further modeling is required to reconcile these experimental results. In addition, thalamocortical interactions are known to be integral to the synchronization of spindles [5, 33]. In our new study, the core thalamocortical system revealed relatively high spindle density produced by focal and strong thalamocortical and corticothalamic projections. Such a pattern of connectivity between core thalamus and middle cortical layers allowed input from a small region of the cortex to initiate and maintain focal spindles in the core system. In contrast, the matrix system had relatively weak and broad thalamocortical connections requiring synchronized activity in broader cortical regions in order to initiate spindles in the thalamus.

We previously reported [5] that (1) within a single spindle event the synchrony of the neuronal firing is higher in the matrix than in the core system; (2) spindle are initiated in the core and with some delay in the matrix system. The overal density of core and matrix spindle events was, however, the same in these earlier models. In the new study we extended these previous results by explaining differences in the global spatio-temporal structure of spindle activity between the core and matrix systems. Our new model predicts that the focal nature of the core thalamocortical connectivity can explain the more frequent occurrence of spindles in the core system as observed in vivo. The strength of core-to-matrix intracortical connections determined the probability of core spindles to “propagate” to the matrix system. In our new model core spindles remained localized and have never involved the entire network, again in agreement with in vivo data.

We observed that the distribution of inter-spindle intervals reflects a non-periodic stochastic process such as a Poisson process, which is consistent with previous data [18, 27]. The state of the thalamocortical network, determined by the level of the intrinsic and synaptic conductances, contributed to the stochastic nature of spindle occurrence. Building off our previous work [21], we chose the intrinsic and synaptic properties in the model that match those in stage 2 sleep, a brain state when concentrations of acetylcholine and monoamines are reduced [37–39]. As a consequence, the K-leak currents and excitatory intracortical connections were set higher than in an awake-like state due to the reduction of acetylcholine and norepinephrine [40]. The high K-leak currents resulted in sparse spontaneous cortical firing during periods between spindles with occasional surges of local synchrony sustained by recurrent excitation within the cortex that could trigger spindle oscillations in the thalamus. Note that this mechanism may be different from spindle initiation during slow oscillation, when spindle activity appears to be initiated during Down state in thalamus [35]. Furthermore, the release of miniature EPSPs and IPSPs in the cortex was implemented as a Poission process that contributed to the stochastic nature of the baseline activity. All these factors led to a variable inter-spindle interval with long periods of silence when activity in the cortex was not sufficient to induce spindles. While it is known that an excitable medium with noise has a Poisson event distribution in reduced systems [41], here we show that a detailed biophysical model of spindle generation may lead to a Poission process due to specific intrinsic and network properties.

Layer IV excitatory neurons have a smaller dendritic structure compared to Layer V excitatory neurons [42]. Direct recordings and detailed dendritic reconstructions have shown large post-synaptic potentials in layer IV due to core thalamic input [42, 43]. We examined the role of thalamocortical and corticothalamic connections in a thalamocortical network with only one cortical layer (S1 Fig). We found that increasing the synaptic strength of thalamocortical and corticothalamic connections both increased the density and duration of spindles, however it did not influence their synchronization (S1A Fig). In contrast, changing fanout led to an increase in spindle density, duration, and synchronization. Furthermore, we examined the impact of thalamocortical and corticothalamic connections individually without applying a synaptic normalization rule (see Methods). We observed that the thalamocortical connections had a higher impact on spindle properties than corticothalamic connections (S1B Fig). In our full model with multiple layers, which included a weight normalization rule and wider fanout of the matrix pathway (based on experimental findings[16]), the synaptic strength of each thalamocortical synapse in the core pathway was higher than that in the matrix pathway. The exact value of the synaptic strength was chosen from the reduced model to match experimentally observed spindle durations, as observed in EEG/MEG and laminar recordings [17].

The simultaneous EEG and MEG recordings reported here and in our previous publications [14, 29] revealed that (a) MEG spindles occur earlier compared to the EEG spindles and (b) EEG spindles are seen in a higher number of the MEG sensors compared to the spindles occurring only in the MEG recordings. This resembles our current findings, in which the number of regions that were spindling in the core system during a matrix spindle was higher than when there was no spindle in the matrix system. Further, the distribution of spindle onset delays between the systems indicates that during matrix spindles some neurons of the core system fired early, and presumably contributed to the initiation of the matrix spindle, while others fired late and were recruited. Taken together, all the evidence suggests a characteristic and complex spatiotemporal evolution of spindle activity during co-occurring spindles, where spindles in the core spread to the matrix and in turn activate wider regions in the core leading to synchronized activation across cortical layers that is reflected by strong activity in both EEG and MEG. Thus, the model predicts that co-occurring spindles could lead to the recruitment of the large cortical areas, which indeed has been reported in previous studies [28, 44]. At the same time, local spindles occurring in the model within deep cortical layers may correspond to the local spindles observed in some studies [45], or may be even hidden from empirical recordings because of their localized and low amplitude properties. Finally, regional differences in thalamocorical and corticocortical connections could explain the characteristic regional and spatial patterns of spindles observed in human recordings [15].

The correspondence of the matrix vs core thalamocortical system to EEG vs MEG recordings was proposed originally to explain the differences between properties of the EEG and MEG spindles [14, 46]. Several lines of evidence support this hypothesis and was reviewed by Piantoni et al [15]. MEG and EEG share neural generators, but differences arise due to the biophysics of their cancellation patterns as they project from cortex to sensor. We have recently applied a biophysical forward model of MEG and EEG generation from a large-scale thalamocortical model that is similar to the model used in this study [47]. As hypothesized, in this combined neural/biophysical model, core-dominant spindles were more MEG-weighted than matrix-dominant spindles.

Human EEG studies have previously reported the existence of two types of spindles based on frequency—slow and fast spindles (9–12 and 12-15Hz). In our study, there was small difference (core-13.8 (subharmonic at 6.7Hz) and matrix-14.4 Hz (subharmonic-7.2Hz)) in the frequency between core and matrix spindles. However, this difference was much smaller than between fast and slow spindles (which are 9–12 and 12-15Hz) reported in vivo. These results are consistent with laminar recordings of spindles in humans [17], where the average frequency of middle vs upper layer spindles does not differ significantly. Furthermore, in intracranial recordings, both slow and fast spindles occur after down states [35, 36], as opposed to the reported occurrence of slow spindles before down states in the EEG [48]. Taken together, these findings suggest that the properties of the thalamocortical and corticothalamic connections explored here are not sufficient to explain the origin of fast vs. slow spindles as observed in rodent studies.

The relationship between spindle phase and spike timing of cortical neurons has been examined by previous studies, though their findings have been contradictory. While some studies have shown a preference in phase [45], others have shown no such preference [49]. In a recently published analysis of spindles recorded in the thalamus and cortex of humans [35], we found that thalamic spindling appears to drive cortical spindling, including both LFP and high gamma activity. This suggests that the nature of thalamic connections could influence the phase preference of spiking during spindle oscillations. In this new study, we measured the phase of cortical neurons’ spiking during spindles (S2 Fig) and we observed that, in our model, neurons both in the core and the matrix systems had a higher preference to spike at the peak of the spindle oscillation (corresponding to the oscillation phase values close to pi or–pi). We also compared the variability of the spiking phase in the matrix pathway versus the core one (S2C Fig). The statistical test comparing the phase of spiking in the core versus matrix systems aggregated for all cortical neurons was not significant (two sample KS test of phase distribution, KS statistic = 0.12, p = 0.44). However, normalized probability of spiking for the different spindle oscillation phases (obtained from the normalized histogram binned at 100 intervals) was significantly different between the core and the matrix for many values of phase. This suggests that the phase of spiking in the matrix pathway has a trend for being more variability than in the core pathway.

Increase in spindle density following learning is a robust experimental finding that suggests a role for sleep in memory consolidation [2, 6, 7, 10, 50, 51]. However, the neural mechanisms that increase spindle density after learning are not known. The hippocampal CA1 region projects to both superficial and deep layers of the rodent prefrontal cortex [52, 53]. In addition, experiments with simultaneous recordings from cortex and hippocampus report that during NREM sleep, the two structures show coordinated activity [54, 55], underlying spike sequence replay and the reactivation of memories that were recently learned while awake [56]. In our study, we found that activation of layer 3/4 of the neocortex triggers spindles that propagate between the core and matrix systems and eventually lead to spindle recruitment in wide regions of the neocortex. Based on these findings, we predict that hippocampal input to superficial cortical layers (layer 2/3/4) during NREM sleep can induce local activation and spindles in the core system, which then propagate to the matrix system thereby activating large cortical regions in both layers, potentially contributign to memory consolidation.

Elevated spindle density may arise due to the changes in the cortical microcircuit, of which excitatory interlaminar connections form the main component [57]. This circuit is implicated as a site of sensory coding and learning [58]. In this study, we identified that connection strength from the core to the matrix, but not *vice verse*, was critical in determining spindle density. This predicts that the increase in spindle density following a hippocampal-dependent task may arise from the strengthening of feedforward projections from middle to superficial cortical layers.

In sum, our study identified a rich set of the local and global network mechanisms involved in the propagation and interactions of spindles across different cortical structures. While spindle activity in the model arises from thalamic circuits, our study supports the idea that thalamocortical and intracortical projections significantly shape the properties of spindling activity and that this may explain the characteristic changes of spindle density associated with sleep-related memory replay.

## Materials and methods

### Ethics statement

The human research reported in this study was approved by the institutional review board at Partners Healthcare Network. Written informed consent was directly obtained from all subjects prior to their participation.

### Computational models

#### Intrinsic currents–thalamus

Conductance based models of thalamocortical neuron (TC) and thalamic reticular neuron (RE) included one compartment:

$$C_{m}\frac{dV}{dt}=–g_{leak}(V–E_{leak})–I_{int}–I_{syn}$$ (1)

where the membrane capacitance, C_m_, is equal to 1 μF/cm^2^, non-specific (mixed Na^+^ and Cl^-^) leakage conductance, g_leak_, is equal to 0.01 mS/cm^2^ for TC cells and 0.05 mS/cm^2^ for RE cells, and the reversal potential, E_leak_, is equal to– 70 mV for TC cells and– 77 mV for RE cells. I_int_ is the sum of active intrinsic currents, and I_syn_ is the sum of synaptic currents. The area of a RE cell and a TC cell was 1.43×10–4 cm^2^ and 2.9×10–4 cm^2^, respectively [26, 59].

Both RE and TC cells include fast sodium current, I_Na_, a fast potassium current, I_K_, a low-threshold Ca^2+^ current I_T_, and a potassium leak current, I_KL_ = g_KL_ (V–E_KL_), where E_KL_ = – 95 mV. In addition, a hyperpolarization-activated cation current, I_h_, was included in TC cells. For TC cells, the maximal conductances are g_K_ = 10 mS/cm^2^, g_Na_ = 90 mS/cm^2^, g_T_ = 2.2 mS/cm^2^, g_h_ = 0.017 mS/cm^2^, g_KL_ = 0.03 mS/cm^2^. For RE cells, the maximal conductances are g_K_ = 10 mS/cm^2^, g_Na_ = 100 mS/cm^2^, g_T_ = 2.3 mS/cm^2^, g_leak_ = 0.005 mS/cm^2^. The expressions of voltage- and Ca^2+^- dependent transition rates for all currents are given in [26, 59].

#### Intrinsic currents–cortex

The cortical pyramidal cells (PY) and interneurons (IN) were represented by two-compartment model with channels simulated by Hodgkin–Huxley kinetics. Each compartment was described by Eq (1) with an additional current from adjacent compartment, I_d,s_ = g_c_(V_d,s_−V_s,d_), where V_d_ (resp. V_s_) is the voltage of the dendritic (resp. axo-somatic) compartment. The axo-somatic and dendritic compartments were coupled by an axial current with conductance g_c_. The resistance (r) between compartments was set to 10 MΩ. The firing properties of the model depended on the coupling conductance between compartments (g_c_ = 1/r) and the ratio of dendritic area to the axosomatic area R (Mainen and Sejnowski, 1996). We used a model of a regular-spiking neuron for PY cells (R = 165) and a model of a fast spiking neuron for IN cells (R = 50).

The PY neurons and INs contained fast Na^+^ channels, I_Na_, with a higher density in the axosomatic compartment than in the dendritic compartment. In addition, a fast delayed rectifier potassium K^+^ current, I_K_, was present in the axosomatic compartment. A persistent sodium current, I_Na(p)_, was included in both axosomatic and dendritic compartments. A slow voltage-dependent non-inactivating K^+^ current, I_Km_, a slow Ca2+-dependent K^+^ current, I_Kca_, a high-threshold Ca^2+^ current, I_HVA_, and a potassium leak current, I_KL_ = g_KL_(V-E_KL_) were included in the dendritic compartment only. The expressions of the voltage- and Ca^2+^-dependent transition rates for all currents are given in Timofeev et al. [60]. For axosomatic compartment, the maximal conductances and passive properties were S_soma_ = 1.0×10–6 cm^2^, g_Na_ = 3000 mS/cm^2^, g_K_ = 200 mS/cm^2^, g_Na(p)_ = 0.07 mS/cm^2^. For dendritic compartment: Cm = 0.75 μF/cm^2^, gL = 0.033 mS/cm^2^, gKL = 0.0025 mS/cm^2^, S_dend_ = S_soma_ × R, g_HVA_ = 0.01 mS/cm^2^, g_Na_ = 1.5 mS/ cm^2^, g_Kca_ = 0.3 mS/cm^2^, g_Km_ = 0.01 mS/cm^2^, g_Na(p)_ = 0.07 mS/ cm^2^, E_KL_ = – 95 mV. E_leak_ was– 68 mV for PYs and– 75 mV for INs [26, 59]. For interneurons, no I_Na(p)_ was included.

#### Synaptic currents

All synaptic currents were calculated according to:

$$I_{\mathrm{syn}}=g_{\mathrm{syn}}[O]\;(V–E_{\mathrm{syn}})$$ (2)

where g_syn_ is the maximal conductance, [O] is the fraction of open channels, and E_syn_ is the reversal potential. In RE and PY cells, reversal potential was 0 mV for AMPA and NMDA receptors, and –70 mV for GABA-A receptors. For TC cells, the reversal potential was –80 mV for GABA-A receptors, and –95 mV for GABA-B receptors. A simple phenomenological model characterizing short-term depression of intracortical excitatory connections was included in the model. According to this, a maximal synaptic conductance was multiplied to a depression variable, D, which represents the amount of available synaptic resources. Here, D = 1 –(1 –D_i_ (1 –U)) exp (–(t–t_i_)/τ) where U = 0.2 was the fraction of resources used per action potential, τ = 500 msec was the time constant of recovery of the synaptic resources, D_i_ was the value of D immediately before the ith event, and (t–t_i_) is the time after i^th^ event.

GABA-A, NMDA, and AMPA synaptic currents were modeled by the first-order activation schemes. Dependence of postsynaptic voltage for NMDA receptors was $1/(1+e^{\frac{V_{post}–V_{th}}{\delta }})$, where V_th_ = – 25 mV, *δ* = 12.5 mV. GABA-B receptors were modeled by a higher-order reaction scheme that considers the activation of K^+^ channels by G-proteins. The equations for all synaptic currents were given in [60]. Spontaneous miniature EPSPs and IPSPs were implemented in the model. The arrival times of miniature EPSPs and IPSPs followed the Poisson process (Stevens, 1993), with time-dependent mean rate μ = (2/(1+exp(-(t-t_0_)/400))-1)/250 where t was real time and t_0_ was timing of the last presynaptic spike occurring.

#### Network geometry

The thalamocortical network model was constructed using layers of one-dimensional network geometry that included cortical pyramidal cells (layer II-IV; layer V; layer VI), cortical interneurons (layer II-IV; layer V; layer VI), thalamocortical neurons (with matrix and core systems) and thalamic reticular neurons. A general connectivity scheme among different populations of neurons is shown in Fig 5. In total, there were 3000 pyramidal neurons (1000 in each population), 400 interneurons (200 in each population), 400 thalamic reticular neurons, and 400 thalamocortical neurons (200 in each system).

The maximal conductances of the connections within cortical layers were (subscript indicates cell types and superscript indicates type of connection) $g_{PY-PY}^{AMPA}$ = 0.6 μS/cm^2^, $g_{PY-PY}^{NMDA}$ = 0.06 μS/cm^2^, $g_{PY-IN}^{AMPA}$ = 0.2 μS/cm^2^, $g_{PY-IN}^{NMDA}$ = 0.08 μS/cm^2^, $g_{IN-PY}^{GABA-A}$ = 0.15 μS/cm^2^. The maximal conductances for connections between layers were: $g_{PY-PY}^{AMPA}$ = 0.3 μS/cm^2^, $g_{PY-PY}^{NMDA}$/cm^2^ = 0.75 μS/cm^2^. Thalamocortical and corticothalamic connections for core and matrix system had the following conductances: $g_{PY-TC}^{AMPA}$ = 0.05 μS/cm^2^, $g_{PY-RE}^{AMPA}$ = 0.15 μS/cm^2^, $g_{TC-PY}^{AMPA}$ = 0.15 μS/cm^2^, $g_{TC-IN}^{AMPA}$ = 0.1 μS/cm^2^. Connections within the thalamus for both the core and matrix system had the following conductances: $g_{RE-RE}^{GABA-A}$ = 0.1μS/cm^2^, $g_{RE-TC}^{GABA-A}$ = 0. 06μS/cm^2^, $g_{RE-TC}^{GABA-B}$ = 0.0025 μS/cm^2^ and $g_{TC-RE}^{AMPA}$ = 0.06 μS/cm^2^. The strength of an individual synapse was defined as a ratio of the total synaptic strength of a connection over the number of individual synaptic inputs that the neuron received from the same type of neurons with the same type of synapses.

To explore how core and matrix spindles interact in the cortex, in this study we intentionally avoided direct propagation of spindles between core and matrix systems within the thalamus. Indeed, a critical *in vivo* spindle property described in this new study–localized core spindles that only occasionally lead to the global spindle in the matrix system–required relative independence of spindle generators in the thalamus. We would like to note that current experimental evidence is not clear on the amount of overlap between the RE thalamic cells corresponding to the specific and non-specific nuclei in the thalamus [61].

### Experimental methods

Extracranial electromagnetic fields were recorded in 4 healthy adults (3 female). Subjects did not report any neurological problems including sleep disorders, epilepsy, or substance dependence. In addition, subjects did not consume caffeine or alcohol on the day of recording. A whole-head MEG system with integrated EEG cap (Elekta Neuromag) was used to collect 204 planar gradiometers and 60 EEG channels. EEG data were referenced to an averaged mastoid. Additional details concerning data collection can be found in [46]. Sleep staging was performed by three neurologists according to standard criteria (Rechtschaffen and Kales, 1968). Data analyzed came from a 17.5 ± 3.4 (mean ± SD) minute period of stage 2 sleep.

Data were acquired at 603.107 Hz. Gross artifacts and bad channels were excluded manually. The continuous data were band-pass filtered to between 0.1 and 30 Hz and ICA (Delorme and Makeig, 2004) was used to remove the ECG component. Spindles were automatically detected in each MEG and EEG channel using a method modified from [45].The 10–16 Hz analytic signal was extracted from the data using the Hilbert transform and the envelope obtained by computing its elementwise modulus. The spindle-band envelope was smoothed with a Gaussian kernel (300 ms width, 40 ms σ). Putative spindles were initially marked as contiguous regions of the smoothed spindle-band envelope where the envelope amplitude was more than 2 standard deviations above the mean. Marked regions were then expanded until amplitude dropped below 1 standard deviation above the mean. Putative spindles shorter than 500 ms and longer than 2 s were excluded from further analysis. Inter-spindle intervals (ISIs) were computed from spindle center to center. Outlying ISIs longer than 20 seconds were excluded, as these are likely caused by false negatives in spindle detection. ISIs from all subjects and all channels were pooled together to form a single distribution for EEG and gradiometer data, respectively.

## Supporting information

S1 Fig Effect of network properties on spindle activity. A. Inter-spindle interval (left), spindle duration (middle) and phase locking across different neuronal groups (right) for different strength and fannout of thalamocortical and corticothalamic connections. Synaptic weights were varied without applying normalization by the number of input connections. B. Density and duration of spindles for different strengths of corticothalamic (left) and thalamocortical (right) connections.
(TIF)

S2 Fig Distribution of the spike phase across all cortical neurons in the core (A) and matrix (B) systems. C. Mean (across all neurons) normalized probability of spiking at different spindle phase values in the core and matrix pathway. Bars indicate standard error. Star (*) indicates p<0.0005 in a 2 sample KS test.
(TIF)
