# Supplementary figures and images for: Thalamocortical and intracortical laminar connectivity determines sleep spindle properties
Source: PLoS Comput Biol. 2018 Jun 27;14(6):e1006171. doi: 10.1371/journal.pcbi.1006171 (PMC6039052; doi:10.1371/journal.pcbi.1006171)

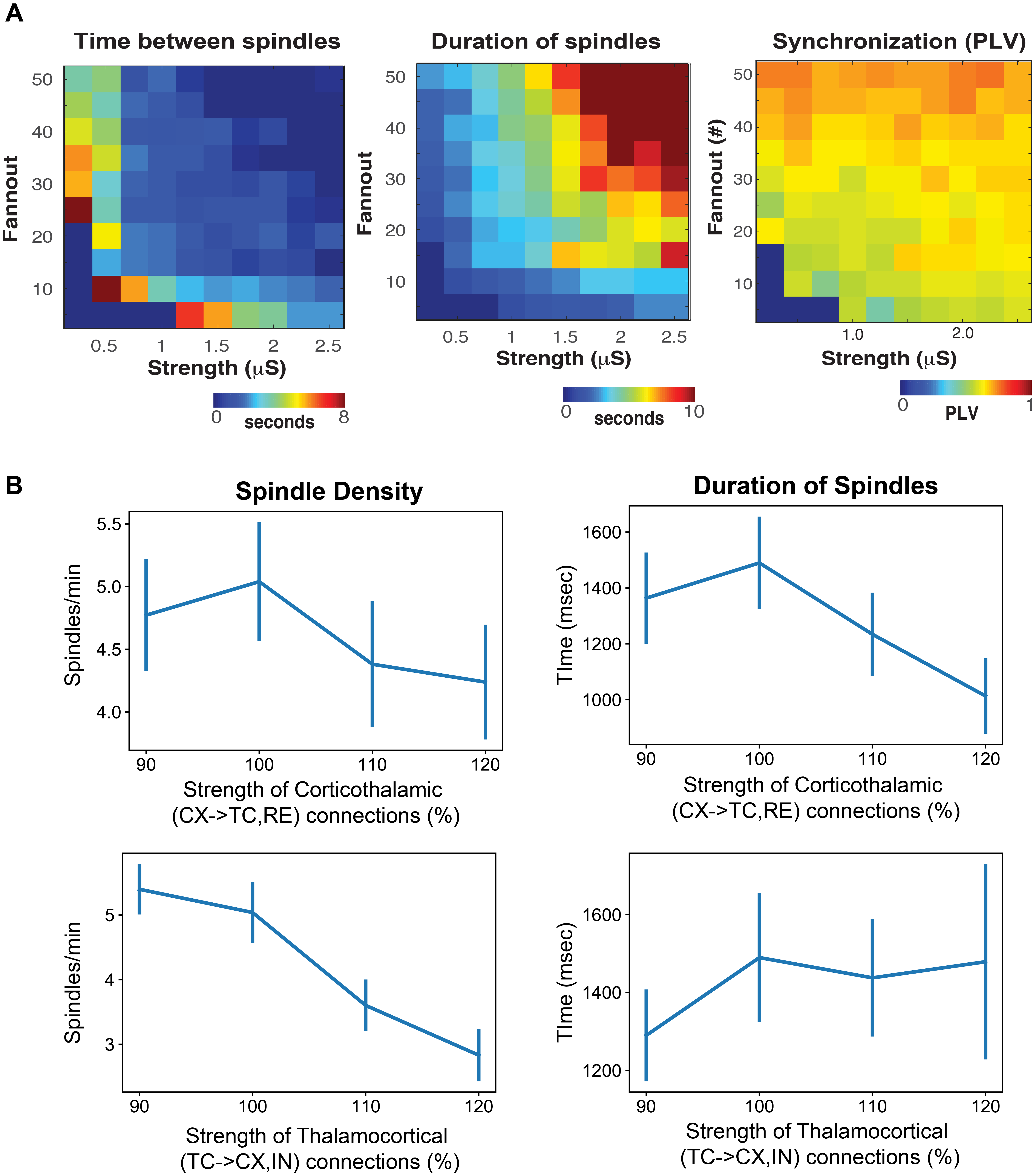

Supplement: S1 Fig — A. Inter-spindle interval (left), spindle duration (middle) and phase locking across different neuronal groups (right) for different strength and fannout of thalamocortical and corticothalamic connections. Synaptic weights were varied without applying normalization by the number of input connections. B. Density and duration of spindles for different strengths of corticothalamic (left) and thalamocortical (right) connections. (TIF) [file pcbi.1006171.s001.tif]

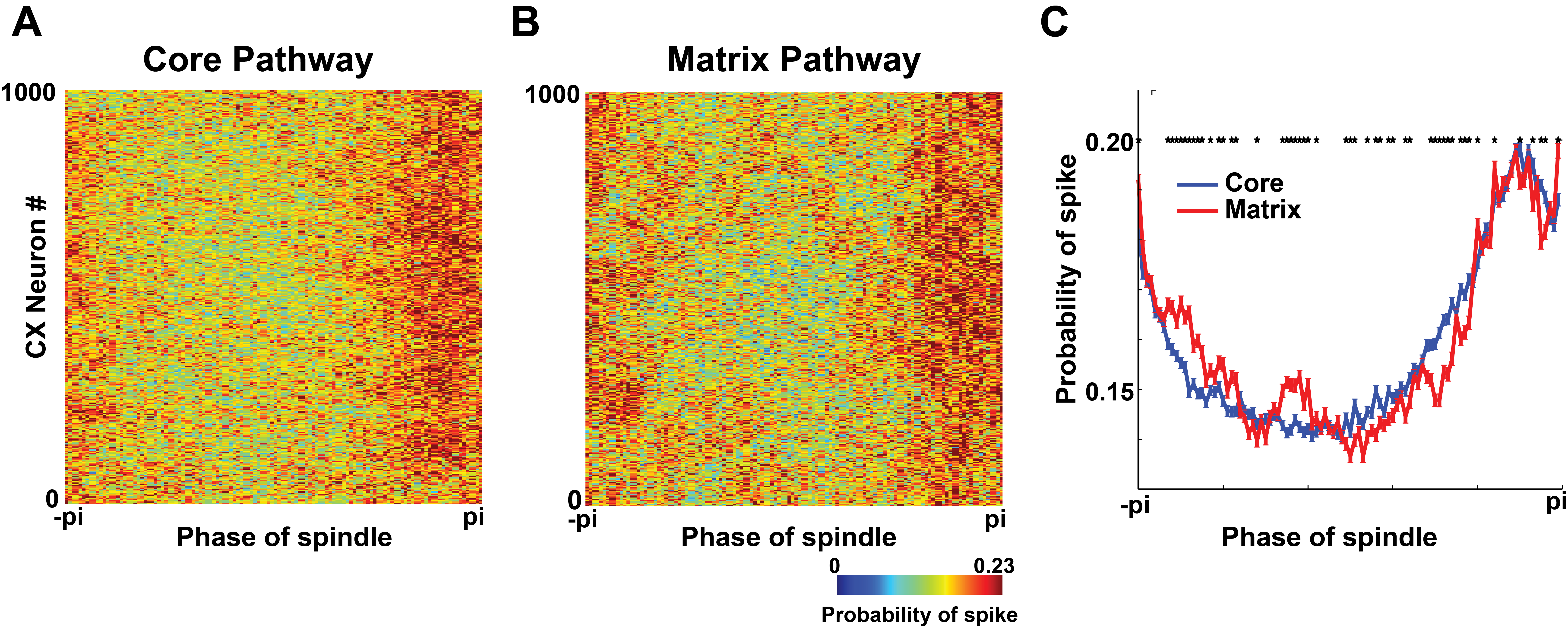

Supplement: S2 Fig — Distribution of the spike phase across all cortical neurons in the core (A) and matrix (B) systems. C. Mean (across all neurons) normalized probability of spiking at different spindle phase values in the core and matrix pathway. Bars indicate standard error. Star (*) indicates p<0.0005 in a 2 sample KS test. (TIF) [file pcbi.1006171.s002.tif]
